# Volumetric and shape analysis of the hippocampus in temporal lobe epilepsy with GAD65 antibodies compared with non-immune epilepsy

**DOI:** 10.1038/s41598-021-89010-z

**Published:** 2021-05-13

**Authors:** Estefanía Conde-Blanco, Saül Pascual-Diaz, Mar Carreño, Emma Muñoz-Moreno, José Carlos Pariente, Teresa Boget, Isabel Manzanares, Antonio Donaire, María Centeno, Francesc Graus, Nuria Bargalló

**Affiliations:** 1 Epilepsy Program, Neurology Department, Hospital Clínic de Barcelona, EpiCARE: European Reference Network for Epilepsy, Institut D’Investigacions Biomèdiques August Pi i Sunyer (IDIBAPS), Carrer de Villarroel, 170, 08036 Barcelona, Spain; 2 Magnetic Resonance Imaging Core Facility, IDIBAPS, Barcelona, Spain; 3 Epilepsy Program, Neuropsychology, Hospital Clínic de Barcelona, Barcelona, Spain; 4 Clinical and Experimental Neuroimmunology Research Team of IDIBAPS, Barcelona, Spain; 5 Epilepsy Program, Neuroradiology Section, Radiology Department, Center of Image Diagnosis (CDIC), Barcelona, Spain

**Keywords:** Neurology, Epilepsy

## Abstract

Glutamic acid decarboxylase 65 antibodies (anti-GAD65) have been found in patients with late-onset chronic temporal lobe epilepsy (TLE). No prior neuroimaging studies have addressed how they affect hippocampal volume and shape and how they relate to cognitive abnormalities. We aimed to investigate both brain structure and function in patients with isolated TLE and high anti-GAD65 levels (RIA ≥ 2000 U/ml) compared to 8 non-immune mesial TLE (niTLE) and 8 healthy controls (HC). Hippocampal subfield volume properties were correlated with the duration of the disease and cognitive test scores. The affected hippocampus of GAD-TLE patients showed no volume changes to matched HC whereas niTLE volumes were significantly smaller. Epilepsy duration in GAD-TLE patients correlated negatively with volumes in the presubiculum, subiculum, CA1, CA2–3, CA4, molecular layer and granule cell-molecular layer of the dentate nucleus. We found differences by advanced vertex-wise shape analysis in the anterior hippocampus of the left GAD-TLE compared to HC whereas left niTLE showed bilateral posterior hippocampus deformation. Verbal deficits were similar in GAD-TLE and niTLE but did not correlate to volume changes. These data might suggest a distinct expression of hippocampal structural and functional abnormalities based on the immune response.

## Introduction

Epilepsies of unknown etiology comprise one-third of the adult epilepsies and represent a significant disease burden^1^. Temporal lobe epilepsy (TLE) is the most common form of epilepsy in adults^2^. Hippocampal sclerosis (HS) is the most common epileptogenic lesion^3^, and the most often treated surgically, with the best results and good seizure control in up to 70% of patients with unilateral HS^4^. In contrast, TLE associated with neuronal antibodies has a worse outcome after surgery as only 16% of patients improve^5^.

An autoimmune etiology should be considered in the diagnostic work-up of TLE as the prevalence of immune mediated TLE may be higher than previously expected. A correct diagnosis is important because immunotherapy will be required in some cases to achieve an optimal seizure control^6^.

Epilepsy associated with antibodies against glutamic acid decarboxylase 65 (anti-GAD65) is the most common cause of autoimmune chronic TLE (GAD-TLE)^7^. Glutamic acid decarboxylase (GAD) catalyzes the decarboxylation of glutamate into γ-aminobutyric acid (GABA) which is the major inhibitory neurotransmitter in the brain^8^. There are 2 major isoforms of GAD, 67kD (GAD-67) and 65kD (GAD-65). The GAD-65 isoform is transiently activated in response to demands for temporary boosts in GABAergic neurotransmission^9^. Low titers of anti-GAD65 may be present in 1–8% of the general population and in patients diagnosed with type 1 diabetes mellitus (T1DM)^8,9^. In contrast, high anti-GAD65 titers have been found in stiff-person syndrome (SPS) and patients with cerebellar ataxia or isolated TLE^10–12^.

Anti-GAD65 were first associated with pharmacoresistant TLE 22 years ago and fewer than 200 cases have since been reported^13^. Series evaluating the hippocampal MRI features of GAD-TLE are scarce^14^ and some of them mix patients with diagnosis of limbic encephalitis and patients with isolated TLE complicating the interpretation of the results^15^. However, to identify specific changes in the MRI of GAD-TLE it would be important to raise the possibility of this diagnosis in the evaluation of TLE and to improve our knowledge on the pathophysiology of the disease.

The aim of the present study was to better characterize the hippocampal morphological changes observed in the MRI of patients with GAD-TLE compared to non-immune mesial TLE (niTLE) and healthy controls (HC) and its relationship to memory deficits.

## Methods

### Patients

We perform a retrospective study of patients with GAD-TLE referred to a tertiary hospital between 2003 and 2017. Eighteen patients were first identified from clinical registries. Eight out of 18 patients fulfilled the following criteria and were finally included: (1) presence of isolated or predominant TLE; (2) high serum levels of anti-GAD65 (≥ 2000 UI/mL) by radioimmunoassay; (3) adequate follow-up in our hospital; (4) MRI available for analysis; and (5) absence of other clinical or MRI findings that could contribute to the epilepsy. The remaining 10 were excluded due to inadequate follow-up, previous history of meningitis, or unavailable 3T MRI. GAD-TLE patients were compared with 8 patients with niTLE and 8 HC matched for age and sex. Inclusion criteria for patients with niTLE were (1) video-EEG diagnosis of mesial temporal lobe epilepsy; (2) febrile seizures; and (3) a latent period, epilepsy onset in mid-to-late childhood. The affected hippocampus was identified by the ictal EEG findings. The study was approved by the Ethics Committee of Hospital Clinic and complies with the ethical standards in accordance with the relevant guidelines and regulations and with the Helsinki Declaration. Informed consent was obtained from all participants in the study.

### Image acquisition and analysis

Scans were performed on a 3T Siemens MAGNETOM TIM Trio scanner (Siemens Medical Systems, Germany), using a 32-channel head coil for radio frequency transmission. Participants had a dedicated epilepsy MR protocol including coronal T2 FLAIR (fluid-attenuated inversion-recovery) and 3D T1-MPRAGE sequences. This last was used for the volumetric and shape analysis described below. 3D T1-MPRAGE acquisitions parameters were TR: 2000 ms, TE: 3 ms, TI: 900 ms, flip angle 9º. Since this was a retrospective analysis, acquisitions with two different voxel sizes were found among the subjects that fulfilled the inclusion criteria: (a) 0.86 × 0.9x0.86, and 192 coronal slices and (b) 0.9 × 1.2x0.9 mm, and 240 coronal slices.

HS was evaluated by an expert neuroradiologist naked eye (NB) as follows: (1) visual atrophy, (2) signal change either in FLAIR or in a T2-weighted sequence, and (3) loss of digitation of the head of the hippocampus.

#### Volumetric analysis

T1-MPRAGE images were evaluated using Free Surfer image analysis suite v6.0 (http://surfer.nmr.mgh.harvard.edu/) to estimate the intracranial volume (ICV), gray matter volume and to automatically segment right and left hippocampal subfields and quantify their volumes^16^. Automatic segmentations were manually checked before running the statistical analysis. Examined hippocampal subfields were the parasubiculum, presubiculum, subiculum, cornu ammonis areas (CA1, CA2-3, CA4), molecular layer of the hippocampus, granule cells and molecular layer of the dentate gyrus (GC-ML-DG), hippocampus-amygdala-transition-area (HATA), fimbria, hippocampal fissure and hippocampal tail^15^. Since contrast between CA2 and CA3 could not be distinguished on MRI they were combined. All volumes were normalized by the total ICV to adjust for differences in head size by using the following equation:

1 $$\text{normalized volume} = \text{raw volume}/\text{ ICV}$$

For volume analysis, volumes were regrouped according to the lateralization of the ictal electroencephalogram (EEG), allowing analyses of the affected and contralateral side to study structural alterations. In GAD-TLE patients with bitemporal epilepsy structural changes both hippocampi were evaluated as affected.

We calculated for all volumes the Z-score using the following formula:

2 $${\text{Z}} = \left( {x -\upmu } \right)/\upsigma$$

where *x* stands for individual normalized volume, μ and σ are the mean and standard deviation of respective normalized volumes in the HC group. Z-scores were used to evaluate the volumetric deviation in both GAD-TLE and niTLE from the HC.

We investigated structural changes in hippocampal subfields and compared the results within subjects and among the three groups. For this we calculated the asymmetry index and the percentage of volumetric differences.

- The asymmetry index (AI) was calculated for each hippocampal subfield using^17^:
  3 $${\text{V}}_{s} = \frac{{V_{s, left} - V_{s, right} }}{{V_{s, left} + V_{s, right} }},$$
  where Vs, left/right refers to the volume of the structures in the left or right hemisphere respectively. Positive scores ≥ 0.2 indicate a leftward asymmetry lateralization (left volume > right volume) and negative scores ≤ − 0.2 indicated rightward asymmetry, that is, the volume of the structure in the right hemisphere was greater than its volume in the left hemisphere. We considered the absolute values to compare differences in asymmetry between groups, regardless of the direction of the difference, using the Kruskal–Wallis one-way analysis of variance.
- The percentage of volumetric difference ($\mathrm{\%}{\mathrm{V}}_{\mathrm{dif}}$) between the affected and the contralateral hippocampus in GAD-TLE and niTLE patients was estimated by the following equation:
  4 $$\% {\text{V}}_{dif} = 100 \times \left( {\frac{{V_{affected} }}{{V_{contralateral} }}} \right),$$
  where $V_{affected}$ and $V_{contralateral} { }$ are the volumes of the structure in the affected and contralateral hemispheres.

#### Shape analysis

We generated 3D-models from the automated segmentation of left and right hippocampus resulting from FSL FIRST^18^ smoothed with a 3 mm Gaussian kernel. Their shape was analyzed using Spherical Harmonics Point Distribution Models (SPHARM-PDM) implemented in the Slicer SALT software^19,20^. Surfaces had 1002 vertices and were aligned to the 3D model generated from the corresponding hippocampus of the MNI reference template. Vertex-level group differences were analyzed using a Multivariate Functional Shape Data Analysis (MFSDA) to test if there were morphological differences between groups^21^.

### Neuropsychological and clinical assessment

Patients with TLE underwent a comprehensive neuropsychological assessment. Verbal memory was assessed with Rey’s Auditory Verbal learning test (AVLT) using total learning (AVLT total) and delayed recall (AVLT recall). Z-scores for each test and for each subject were calculated based on the normative control group means and standard deviations^22^. We defined severely impaired verbal learning if AVLT score was lower than 45, moderately impaired encoding between 45 and 53, and slightly impaired between 54 and 63. Subsets of Logical Memory I and II of the Wechsler Memory Scale-3rd Edition were used to study immediate and delayed recall of two short stories. Visual Reproduction I and II subsets of the Wechsler Memory Scale-Revised were used for the evaluation. Naming functions were evaluated through the Boston Naming Test. A variety of executive functions were measured by the Trail Making Test (TMT).

### Statistical analysis

Demographic data and volumetric findings are reported as n (%) or median (Me) and interquartile range (IQR). To check the impact of TLE etiology in hippocampal volume and subfield volumes, we evaluated differences between GAD-TLE, niTLE and HC using Kruskal–Wallis one-way analysis of variance test [$\chi$^2^(2)] followed by post hoc analysis with Bonferroni correction [t].

Spearman correlations were performed to establish the association between hippocampal subfield volumes with epilepsy duration and neuropsychological memory performance. Differences in age at MR scan, age at epilepsy onset, sex, hand dominance, autoimmune comorbidity, education, the laterality of TLE, presence of HS, seizure frequency, seizure type, and number of antiseizure medication were evaluated using chi-square test or Wilcoxon–Mann–Whitney test when appropriate. Calculations were done in Stata (v14; StataCorp LLC, Texas).

Statistically significant differences in shape analysis were assessed using a Multivariate Functional Shape Data Analysis (MFSDA), including false discovery rate (FDR) to control for multiple comparisons^17^. Null hypothesis was rejected for corrected p-values lower than 0.05.

### Ethical publication statement

We confirm that we have read the Journal’s position on issues involved in ethical publication and affirm that this report is consistent with those guidelines.

## Results

Eight patients with GAD-TLE [median age (IQR)] 37 years (34–40); 6 females), 8 patients with niTLE (37 years (23–52); 7 females) and 8 HC (34 years (31–39 years) 5 females) were included for the analysis. Median epilepsy age of onset was significantly different between GAD-TLE: 29 years (26–31) vs niTLE: 9 years (3–14) (*p* = 0.01). Median duration of epilepsy was significantly different in GAD-TLE with respect to niTLE, 8 years (3–12) compared to niTLE 17 years (13–37) [z = 2.52; *p* = 0.01]. Status epilepticus at onset was present in two GAD-TLE patients and one niTLE patient. Half of the GAD-TLE patients had T1DM, one had hypothyroidism, one had alopecia areata and psoriasis, one had pernicious anemia and one celiac disease and vitiligo (Table 1).

**Table 1:** Demographic, clinical and MRI features of the three groups evaluated.

Characteristics	GAD-TLE (8)	niTLE (8)	HC (8)
Median age at MRI (IQR) (years)	37.3 (5.9)	37.1 (30)	34 (8.5)
Median age at onset (IQR) (years)	29 (5.5)	9 (11.5)	N/A
Duration of Epilepsy Me (IQR)	7.9 (8.6)	16.9 (24.7)	N/A
**Sex (n; %)**			
Female	6 (75)	7 (87.5)	5 (62.5)
Male	2 (25)	1 (12.5)	3 (37.5)
Autoimmune comorbidities n (%)	6 (54.5)	0	0
**Education n (%)**			
Primary education	1 (12.5)	4 (50%)	N/A
Secondary education	1 (12.5)	2 (25%)	
University education	6 (75)	2(25%)	
**Handedness n (%)**			
Right dominance	8 (100)	7 (87.5)	8 (100)
**Hippocampal sclerosis n (%)**	3 (37.5)	8 (100)	
Right	1	1	N/A
Left	0	7	
Bilateral	2	0	
**Type of TLE n (%)**			
Unilateral			
Right	2 (25)	2 (25)	
Left	4 (50)	6 (75)	N/A
Bilateral	2(25)	0 (0)	
**Seizure frequency at onset n (%)**			
Daily	3 (37.5)	0	N/A
Weekly	2 (25)	1 (12.5)	
Monthly	3 (37.5)	7 (87.5)	
**Seizure type**			
FAS and FIAS	7 (87.5)	8 (100)	N/A
FBTCS	1 (12.5)	0 (0)	
Present ASM median (IQR)	3 (1)	3 (2)	N/A
Prior ASM median (IQR)	4.5 (1.5)	2.5 (1)	N/A

*TLE* temporal lobe epilepsy, *FAS* focal aware seizures, *FIAS* focal impaired awareness, *FBTCS* focal to bilateral tonic–clonic seizure, *ASM* antiseizure medication, *Me* median, *IQR* interquartile range, *N/A* not applicable.

### Neuropsychological tests

Intelligence scores of GAD-TLE patients ranged from above average to low average: 5 patients average, 2 above average and 1 low average. All patients showed a tendency to bitemporal dysfunction ranging from mild to severe. Verbal learning encoding and delayed recall were similarly impaired in both GAD-TLE and niTLE despite the side of the epileptic focus. We found severely impaired encoding in 6 (75%) of GAD-TLE patients and 7 (87.5%) of niTLE patients. Executive function and attention were impaired in 1 out of 8 GAD-TLE patients. Visuoconstructive skills and language were mostly conserved in both groups (supplementary Table 2).

### MRI visual analysis

By visual analysis, HS was present in three patients with GAD-TLE (37.5%), one had left HS and two had bilateral HS; three GAD-TLE patients had subtle left hippocampal volume loss on the visual exam and two GAD-TLE patients showed no signs of hippocampal atrophy. In the niTLE group, six patients had left HS and two patients showed right HS.

### Volumetric analysis

**Figure 1:**
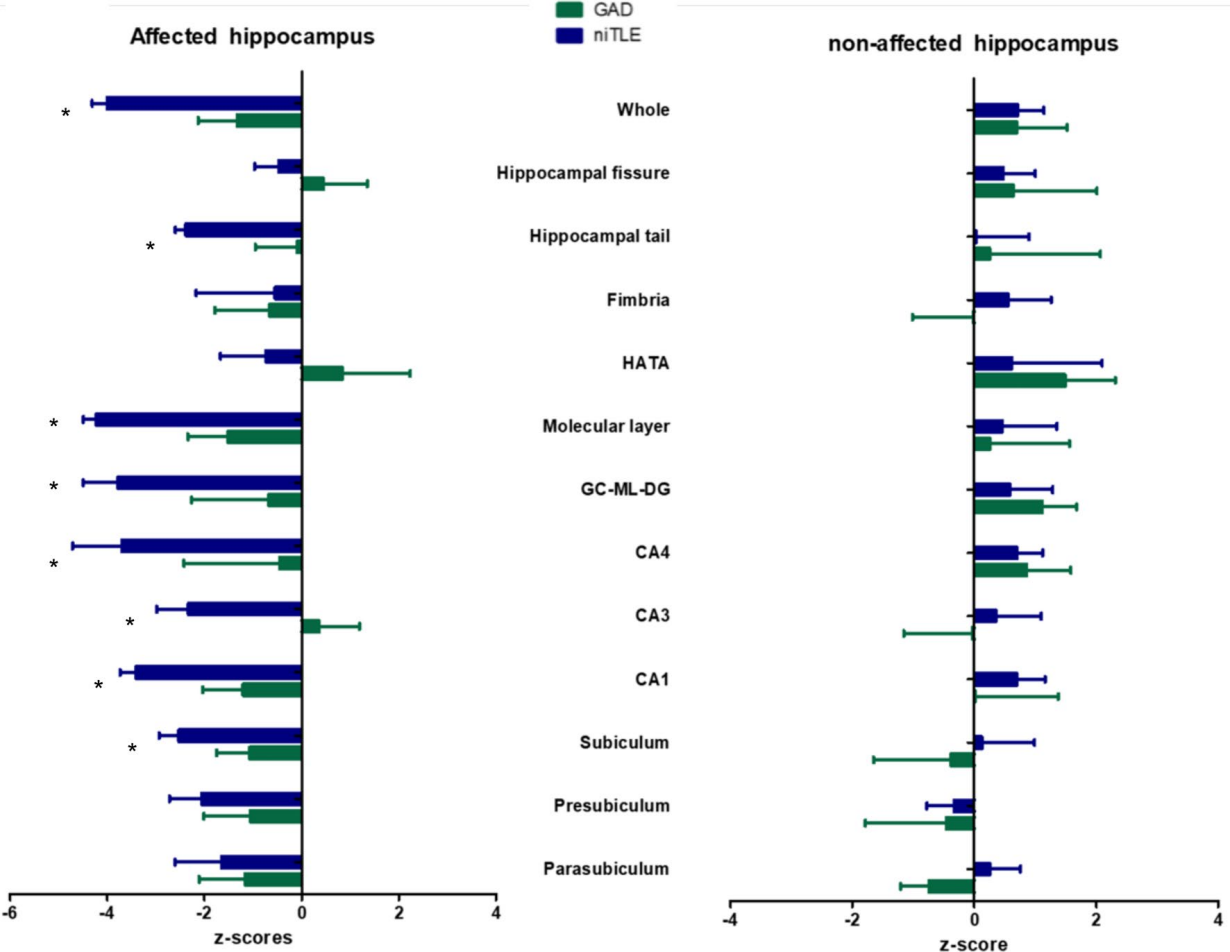
Z-scores of hippocampal subfields volume deviation from HC of each subfield in the affected and contralateral hippocampus of GAD-TLE and niTLE (Median; IQR).

All the volumetric data is showed in Table 2 and supplementary Table 1 and Fig 1. Z-scores of GAD-TLE and niTLE patients are shown in Fig 1.

**Table 2:** Whole and subfield volumes of the affected and contralateral hippocampus of GAD-TLE patients compared with those of niTLE and HC.

Hippocampal Subfields	Proportion between (%)		Affected hippocampus (volume, mm^3^)							Contralateral hippocampus			
	Affected/non-affected hippocampus		GAD-TLE (n:10)† [Me, IQR]	niTLE (n:8)† [Me, IQR]	HC (n:16)† [Me, IQR]	Kruskal wallis test (*p*)	Bonferroni post hoc			GADTLE (n:6)†[Me, IQR]	niTLE (n:8)†[Me, IQR]	Bonferroni post hoc	
	GAD65ab	niTLE					GAD65ab vs niTLE	GAD65ab vs HC	niTLE vs HC			GAD65-niTLE	niTLE-HC
Presubiculum	− 8.9	− 25.2	174.4 (85.10)	146.0 (35.0)	198.8 (51.1)	0.003	NS	NS	< 0.0001*	191.39 (78.89)	195.18 (38.5)	NS	NS
Parasubiculum	− 7.3	− 28.3	34.0 (12.12)	30.9 (10.6)	41.7 (10.2)	0.02	NS	NS	0.016*	36.7(9.08)	43.13(8.59)	NS	NS
Subiculum	− 8.35	− 30.6	251.1(96.19)	202.1 (27.0)	274.3 (49.8)	0.0004	0.004*	NS	< 0.0001*	273.95 (93.89)	291.09 (36.05)	NS	NS
CA1	− 11.7	− 36.9	372.9(159.28)	284.0 (55.8)	429.8 (58.1)	0.0006	0.002*	NS	< 0.0001*	422.27 (107.6)	450.11 (31.86)	NS	NS
CA3	5.2	− 33.9	142.4 (41.07)	94.0 (28.7)	134.5 (28.7)	0.0013	0.001*	NS	0.001*	135.45 (44.46)	142.39 (27.50)	NS	NS
CA4	− 10.4	− 35.5	164.1 (53.47)	116.6 (18.6)	170.6 (20.9)	0.0003	0.001*	NS	0.001*	183.20(48.35)	180.9 (12.71)	NS	NS
GC-ML-DG	− 13.9	− 35.3	186.6 (59.64)	134.4 (20.8)	198.4 (25.3)	0.0003	0.001*	NS	< 0.0001*	216.73 (61.41)	207.75 (16.0)	NS	NS
HATA	− 6.83	− 15.5	42.1 (10.97)	34.7 (8.1)	38.8 (6.4)	NS	NS	NS	NS	45.22 (14.14)	41.05 (9.64)	NS	NS
Fimbria	− 11.44	− 17.6	49.3 (24.96)	50.4 (19.4)	53.7 (12.8)	NS	NS	NS	NS	55.67(24.87)	61.14 (13.64)	NS	NS
Molecular layer	− 13.88	− 36.1	336.1(113.34)	253.2 (45.7)	392.6 (47.5)	0.0002	0.001*	NS	< 0.0001*	390.27 (101.29)	396.49 (40.85)	NS	NS
Hippocampal fissure	− 3.32	− 16.5	95.9 (26.7)	80.5 (16.3)	84.2 (24.1)	NS	NS	NS	NS	99.24 (37.28)	96.53 (18.70)	NS	NS
Hippocampal tail	− 4.27	− 30.7	375.5 (83.48)	264.2 (24.9)	372.5 (41.1)	0.0011	0.002*	NS	< 0.0001*	392.23 (123.53)	381.56 (91.66)	NS	NS
Whole Hippocampus	− 15.31	− 35.7	2070.70 (682.0)	1574.1(276.3)	2292.7 (206.1)	0.0003	0.001*	NS	< 0.0001*	2445.12 (596.72)	2446.75 (230.72)	NS	NS

^**†**^n number of hippocampus included in the analysis, *CA1-2–3-4* Cornu ammonis areas 1,2–3,4, *GC-ML-DG* granule cells in the molecular layer of the dentate gyrus, *HATA* hippocampus-amygdala-transition-area. *NS* non-significant, *Me* median, *IQR* interquartile range.

Patients with niTLE showed smaller volume in the affected hippocampus compared to HC and GAD-TLE patients [$\chi$^2^ (2) = 16.12, *p* = 0.0003].

- Hippocampal subfield volume comparison between GAD-TLE and HC.
  No statistical differences were found in the affected hippocampus volume of these two groups.
  Z-score analysis GAD-TLE patients showed that affected hippocampus had z-scores volumes close to the mean of HC: presubiculum − 1.05 (IQR 3.0), parasubiculum − 1.16 (IQR 1.88), subiculum − 1.10 ( IQR: 2.9); CA1 − 1.20 ( IQR 3.9) , CA3 0.3 (IQR 2.3) ; CA4 − 0.45 (IQR 3.7), GM-ML-DG − 0.67 (IQR 3.5), molecular layer − 1.51 (3.70) , fimbria − 0.66 (IQR 2.55), HATA 0.84 (IQR 2.31), hippocampal tail − 0.09 (1.72), and hippocampal fissure − 0.47 (0.97).
  No differences in the contralateral hippocampus were found between these two groups.
- Hippocampal subfield volume comparison between niTLE and HC.
  We found significant differences in parasubiculum [t = 3.00, *p* = 0.016], presubiculum [t = 4.13, *p* = 0.001], subiculum [t = 5.23, *p* < 0.0001], CA1 [t = 4.98, *p* < 0.0001], CA2–3 [t = 4.26, *p* = 0.001], CA4 [t = 5.33, *p* < 0.0001], GC-ML-DG [t = 5.33, *p* < 0.0001], molecular layer (t = 5.66, *p* < 0.0001) and hippocampal tail [t = 4.48, *p* < 0.0001] of the affected hippocampus (Figure S1, supplementary).
  Z-score analysis showed that patients with niTLE had a significant decline in parasubiculum − 1.64 (1.65), presubiculum − 2.05 (1.23), subiculum − 2.53 (IQR 0.80), CA1 − 3.4 (IQR 1.3), CA2–3 − 2.33 (IQR: 1.23), CA4 − 3.70 (IQR 1.27), GC-ML-DG: − 3.78 (IQR: 1.24), molecular layer − 4.22 (IQR 1.49) and hippocampal tail − 2.39 (IQR 0.51).
  No differences in the contralateral hippocampus were found between these two groups.
- Hippocampal subfield volume comparison between GAD-TLE and niTLE.
  Patients with niTLE showed smaller volume in the affected hippocampus compared GAD-TLE patients [$\chi$^2^(2) = 16.12, *p* = 0.0003]. The hippocampal regions that show significant differences between niTLE and GAD-TLE were subiculum (t = 3.50, *p* = 0.004), CA1 (t = 3.82, *p* = 0.002), CA3 (t = 3.95, *p* = 0.001), CA4 (t = 4.18, *p* = 0.001), GC-ML-DG (t = 4.18; *p* = 0.001), molecular layer (t = 4.28, *p* = 0.001) and hippocampal tail (t = 3.83, *p* = 0.002).
  No differences in the contralateral hippocampus were found between these two groups.

### Asymmetry index

**Figure 2:**
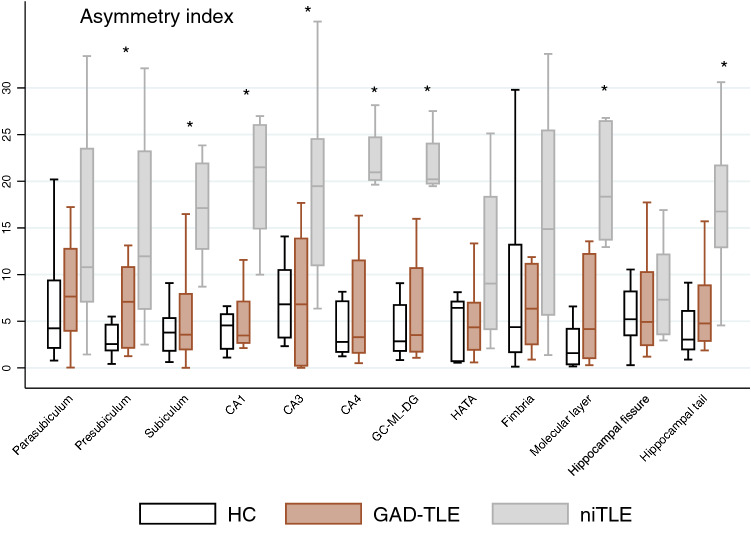
Asymmetry index: significant asymmetry towards the contralateral hippocampus in GAD-TLE compared to niTLE and HC. *Significant asymmetry in each subfield (Kruskal–Wallis test).

The asymmetry index revealed differences in the AI of the whole hippocampus between the 3 groups [$\chi$^2^(2) = 7.0, *p* = 0.004] and in the following hippocampal subfields values: presubiculum [$\chi$^2^(2) = 8.23, *p* = 0.016], subiculum [$\chi$^2^(2) = 10.58, *p* = 0.005], CA1[$\chi$^2^(2) = 14.9, *p* = 0.006], CA3 [$\chi$^2^(2) = 7.60, *p* = 0.022], CA4[$\chi$^2^(2) = 14.50, *p* < 0.001], GC-ML-DG[$\chi$^2^(2) = 14.54, *p* < 0.001], molecular layer [$\chi$^2^(2)15.43, *p* < 0.001] and hippocampal tail [$\chi$^2^(2) = 12.7, *p* = 0.002] (Fig. 2).

The AI of GAD-TLE patients compared to niTLE revealed a significantly asymmetry in subiculum 3.55 (IQR 5.95) versus 17.13 (IQR 9.16) [t = 4.34, *p* = 0.001], CA1 3.47 (IQR 4.45) versus 21.50 (IQR 11.10) [t = 5.29, *p* < 0.0001], CA3 6.81 (IQR 13.63) versus 19.50 (IQR 13.53) [t = 2.77, *p* = 0.031], CA4 3.29 (IQR 9.90) versus 20.96 (IQR 4.58) [t = 4.94, *p* < 0.0001], GM-ML-DG 3.52 (IQR 8.96) versus 20.22 (IQR 4.29) [t = 4.90, *p* < 0.0001], molecular layer 4.17 (IQR 11.18) versus 18.34 (IQR 12.71) [t = 4.95, *p* < 0.0001] and hippocampal tail 4.77 (IQR 5.96) versus 16.76 (IQR 8.77) [t = 3.54, *p* = 0.006].

GAD-TLE and HC showed no significant differences according to AI. niTLE showed significant asymmetry in the subfields that were significantly different compared to GAD-TLE in addition to the presubiculum (Supplementary text).

### Correlation between hippocampal volume and duration of epilepsy and neuropsychological tests

**Figure 3:**
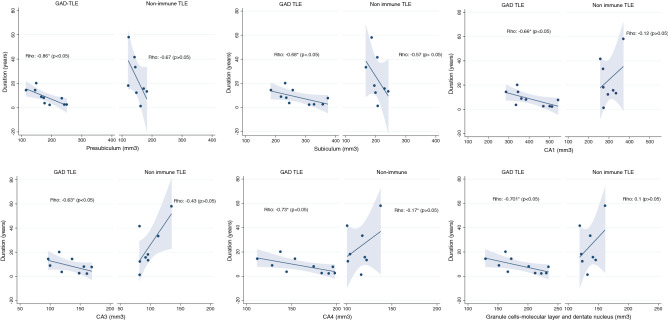
Hippocampal subfields and correlation with disease duration. In the plot the dots represent values for each hippocampal subfield and its correlation with epilepsy duration. On GAD-TLE patients we see a strong negative correlation in the represented subfields, while in niTLE volumes appear randomly scattered and therefore show a non-significantly weak or absent correlation. *CA1–2–3–4* cornu ammonis areas 1,2–3,4, *GC-ML-DG* granule cells in the molecular layer of the dentate gyrus, *HATA* hippocampus-amygdala-transition-area.

Significant correlations between the duration of the disease and volume in some hippocampal subfields were found for the GAD-TLE patients but not for the niTLE. The presubiculum [Rho = − 0.860, *p* = 0.001], subiculum [Rho = − 0.680, *p* = 0.032], CA1 [Rho = − 0.665, *p* = 0.036], CA2–3 [Rho = − 0.634, *p* = 0.049], CA4 [Rho = − 0.732, *p* = 0.016], GC-M-DG [Rho = − 0.701, *p* = 0.024] and molecular layer [Rho = − 0.756, *p* = 0.011] were smaller in GAD-TLE patients with longer duration of the epilepsy (Fig. 3).

We found no correlation between adjusted hippocampal subfield volume and scores for immediate verbal learning, delayed recall, logical memory, and visual memory in GAD-TLE patients. In niTLE patients verbal retrieval score correlated to higher volumes in parasubiculum, subiculum and CA1 of the right hippocampus (supplementary table 3).

### Shape analysis

**Figure 4:**
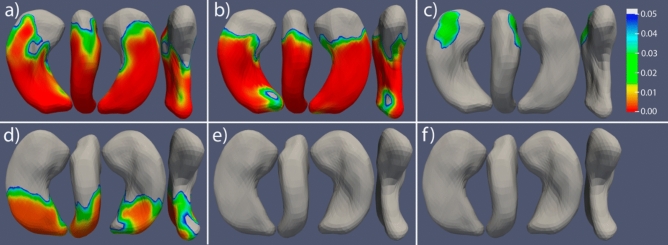
Shape comparison of the respective ipsilateral and contralateral hippocampus in patients with left GAD-TLE compared to left niTLE and HC. (**a**) Left hippocampus of niTLE versus HC; (**b**) left hippocampus of niTLE versus GAD-TLE; (**c**) left hippocampus of GAD-TLE versus HC; (**d**) right hippocampus of niTLE versus HC; (**e**) right hippocampus of niTLE versus GAD-TLE; (**f**) right hippocampus of GAD-TLE versus HC. Thresholded scale on the right indicates FDR corrected *p* values of significant shape change–cooler colors indicate lesser significance and warmer colors significant change.

Due to sample size limitations, patients were only included in the shape analysis if they had the left hemisphere affected (both unilateral and bilateral affectation). This results in 6 GAD-TLE, 6 niTLE and 8 controls included in this analysis. Shape analysis showed a deformation circumscribed to the head of the affected hippocampus in GAD-TLE patients whereas a severe deformation, mainly in the posterior part, of the affected and contralateral hippocampi was observed in niTLE patients compared to HC (Fig. 3). Nevertheless, the differences in the affected hippocampus are observed in bigger areas than in the contralateral hemisphere. There were no significant differences in the shape of the contralateral hippocampus between GAD-TLE patients and those of the other two groups (Fig. 4).

## Discussion

The main finding of our study is that, while cognitive impairment is similar in niTLE and GAD-TLE patients, changes in hippocampal morphology of GAD-TLE subjects are much more subtle than in niTLE. Hippocampal asymmetry indexes were different in GAD-TLE and niTLE with a higher magnitude of asymmetry in the latter. Despite no significant differences between GAD-TLE and HC were detected in hippocampal subfield volumes, vertex-wise comparison of hippocampal shape showed significant differences in the hippocampal shape of GAD-TLE patients in comparison to HC circumscribed to a small area in the head of the affected hippocampus. However, niTLE patients showed widespread deformation, mainly in the posterior part, of both affected and contralateral hippocampus. Our study highlights the fact that volumetric changes might not capture the functional damage observed in the neuropsychological evaluation. Detailed quantitative studies to evaluate the possible atrophic changes may be more accurate and robust than the visual qualitative measurements.

Hippocampal volume correlated with duration of epilepsy in GAD-TLE, mainly in presubiculum, CA4 and molecular layer. No significant correlation was found in niTLE patients which suggests a potential different pathophysiological mechanism that could involve a one-time event in niTLE captured by uniform volume loss in different hippocampal subfields. In GAD-TLE, the time course and type of pathologic changes might be slower, and with a distinctive regional vulnerability of the hippocampal subfields that could selectively damage certain hippocampal subnetworks and their performance^23^. In order to characterize the presumed distinct vulnerability, we selected patients with niTLE who had a history of precipitating trigger before age of 5, early seizure onset, because they were associated with HS type 1 from the ILAE^2^. Contrarily, pathology reports in GAD-TLE patients found predominant neuronal cell loss and gliosis in CA4 and the dentate gyrus^24^, associated to HS type 3, and pointed to less favorable surgical outcome^25^. So far HS ILAE type 2 and 3 have not been systematically studied and much is left to learn.

Hippocampal shape analysis is able to capture hippocampal morphology which is not represented by global or regional volume measurements. Our results show a significant difference between the average-shape of the anterior affected hippocampus in GAD-TLE patients compared to HC which may imply functional deterioration that is not captured by the volumetric analysis and differs from niTLE. niTLE shows alteration of the posterior hippocampus in both sides compared with HC. A recent study associated significant shape surface alterations in the left hippocampal head with a higher risk of poor verbal memory^26^. Worsened verbal memory after left anterior temporal lobe removal was also predicted by atrophy of the left hippocampal tail^27^. Mesial temporal lobe structures, particularly, the hippocampus is one element in the widespread networks of cortical and subcortical brain structures supporting declarative and episodic memory functions^28,29^. We hypothesize that chronic niTLE patients who had a history of precipitating trigger before age of 5 and early seizure onset had a higher degree of brain plasticity and could have undergone a functional reorganization of the entire hippocampal circuitry preserving memory functions to some extent^30^. We presume that changes in hippocampal tail shape may be associated with functional reorganization of the entire hippocampal circuitry in chronic niTLE. Differences in the tail shape were not observed in GAD-TLE patients because of a potentially different pathophysiology^25^.

There are scarce studies on hippocampal morphological changes in GAD-TLE compared to other types of TLE and its association to neuropsychological deficits. In our study, GAD-TLE patients presented predominantly with verbal memory encoding and retrieval deficits but with a tendency to bitemporal dysfunction. Attention and flexibility were impaired in 1/8 patients. We only found one prior study by Falip et al., that reported memory impairment in 61% of GAD-TLE patients, defining memory impairment if 1 subtest z-score was 1 SD below the general level of intelligence^31^.

According to previous research^25^, patients with HS ILAE type 2 demonstrate better preoperative verbal memory performance than those with more widespread cell loss in the CA1, CA3, and CA4 subfields. In our study, patients with GAD-TLE showed similar scores in verbal encoding and retrieval to niTLE even when median volumes of hippocampal subfields were similar to HC. Experimental data suggest that CA3 and dentate gyrus granule cells play a major role in memory acquisition, whereas hippocampal CA1 neurons are implicated in place memory and autobiographical memory retrieval. CA3 has been specifically associated to binding promotion during encoding and pattern completion during retrieval^32^. CA4 was involved in declarative memory acquisition. This could favor the model of how memory processing can be organized amongst hippocampal subfields^28,33^.

The pathophysiology of GAD-TLE is unknown. Anti-GAD65 could disrupt the glutamate-GABA balance to one favoring an accumulation of glutamate and reduction in GABA, leading to increased neuronal excitation and seizures^34,35^. Nevertheless, the intracellular location of GAD casts doubts that GAD antibodies are pathogenic. Alternatively, GAD antibodies could be a biological marker of a more complex immune response against GAD that could include T-cells or concurrent antibodies against neuronal surface antigens. So far, there is no standard treatment for immune epilepsy or autoimmune encephalitis^6,36,37^. Duration of GAD-TLE was associated with volume reduction in certain hippocampal subfields. Rapid immunotherapy in addition to antiseizure medication may have a potential effect in slowing atrophy progression. New neurostimulation devices may be useful for seizure control in refractory epilepsy cases^38^.

Our work has limitations, this is a retrospective study and confounders may be present. Sample size is low but considering the low prevalence of GAD-TLE this is a very homogeneous and well-studied group of patients. Another limitation is the unavailability of a proper control group, therefore patients with niTLE have double duration of the disease. Due to this relatively small sample size there could have been a reduced power to detect significant results. We lack histopathological confirmation since patients did not undergo surgical treatment because of poor outcome.

## Conclusion

Our results show that patients with GAD-TLE, present alterations in hippocampal morphology in comparison with HC, only detected by advanced vertex-wise shape analysis methodology, while more extensive shape and volumetric changes were observed in niTLE patients. A correlation with duration of the disease and volume loss in GAD-TLE patients suggest that atrophy may occur progressively over the years and leave a window for therapies before the atrophy is established. GAD-TLE patients showed similar degrees of cognitive impairment and seizure burden as niTLE suggesting that these are more dysfunctional than structural, possibly mediated by some factor related to the inflammatory component. TLE has distinct etiologies and subregionally-specific morphologic and volumetric changes could be relevant as a marker of its specific pathological identity. Future studies including larger multicentric samples are necessary to consider establishing the use of MR imaging to have more detailed information of pathologic entities.

## Supplementary Information

Supplementary Information

## Data Availability

The datasets analyzed during this study are available from the corresponding author upon reasonable request and after approval by institutional authorities.
