# Author Correction: Volumetric and shape analysis of the hippocampus in temporal lobe epilepsy with GAD65 antibodies compared with non-immune epilepsy

**DOI:** 10.1038/s41598-021-98188-1

**Published:** 2021-09-09

**Authors:** Estefanía Conde-Blanco, Saül Pascual-Diaz, Mar Carreño, Emma Muñoz-Moreno, José Carlos Pariente, Teresa Boget, Isabel Manzanares, Antonio Donaire, María Centeno, Francesc Graus, Nuria Bargalló

**Affiliations:** 1 Epilepsy Program, Neurology Department, Hospital Clínic de Barcelona, EpiCARE: European Reference Network for Epilepsy, Institut D’Investigacions Biomèdiques August Pi i Sunyer (IDIBAPS), Carrer de Villarroel, 170, 08036 Barcelona, Spain; 2 Magnetic Resonance Imaging Core Facility, IDIBAPS, Barcelona, Spain; 3 Epilepsy Program, Neuropsychology, Hospital Clínic de Barcelona, Barcelona, Spain; 4 Clinical and Experimental Neuroimmunology Research Team of IDIBAPS, Barcelona, Spain; 5 Epilepsy Program, Neuroradiology Section, Radiology Department, Center of Image Diagnosis (CDIC), Barcelona, Spain

Correction to: *Scientifc Reports* https://doi.org/10.1038/s41598-021-89010-z, published online 13 May 2021

The Funding section in the original version of this Article was incomplete.

“This work was sponsored by the Instituto de Salud Carlos III (ISCIII) through the Plan Estatal de Investigación Científica y Técnica y de Innovación 2013 2016, project reference number FIS PI17/01211. ECB was supported by fellowship grant RH041910.”

now reads:

“This work was sponsored by the Instituto de Salud Carlos III (ISCIII) through the Plan Estatal de Investigación Científca y Técnica y de Innovación 2013-2016, project reference number FIS PI17/01211 and the European Regional Development Fund (FEDER). ECB was supported by fellowship grant RH041910.”

The original Article has been corrected.

