# Supplementary material for: Volumetric and shape analysis of the hippocampus in temporal lobe epilepsy with GAD65 antibodies compared with non-immune epilepsy
Source: Sci Rep. 2021 May 13;11:10199. doi: 10.1038/s41598-021-89010-z (PMC8119423; doi:10.1038/s41598-021-89010-z)
Supplement: Supplementary file 1 — Supplementary Information [file 41598_2021_89010_MOESM1_ESM.docx]

**Supplementary Material**

**Title: Volumetric and shape analysis of the hippocampus in temporal lobe epilepsy with GAD65 antibodies compared with non-immune epilepsy**

**Authors: Estefanía Conde-Blanco^1^; Saül Pascual-Díaz^2^; Mar Carreño^1^; Emma Muñoz-Moreno^2^; José Carlos Pariente^2^; Teresa Boget^3^; Isabel Manzanares^1^; Antonio Donaire^1^ María Centeno^1^; Francesc Graus^4^; Nuria Bargalló^2,5^**

1 Neurology, epilepsy program. Hospital Clínic de Barcelona. EpiCARE: european Reference Network for Epilepsy. Institut d'Investigacions Biomèdiques August Pi i Sunyer (IDIBAPS)

2 Magnetic Resonance Imaging Core Facility. IDIBAPS

3. Neuropsychology, epilepsy program. Hospital Clínic de Barcelona.

4. Clinical and Experimental Neuroimmunology research team of IDIBAPS.
5. Neuroradiology section, Radiology department, Center of Image Diagnosis (CDIC) epilepsy program.

Corresponding author: Estefanía Conde-Blanco

Epilepsy program. Department of Neurology. Hospital Clínic de Barcelona, Carrer de Villarroel, 170, 08036 Barcelona. Email: [econdeb@clinic.cat](mailto:econdeb@clinic.cat)

**Methods**

**Imaging analysis**

**a) Volumetric analysis**

Whole hippocampal volumes and subfield volumes were compared between the GAD-TLE epilepsy, niTLE and HC. Hippocampal subfields were analyzed as independent variables.

We investigated the following effects:

**1.** Differences between the volumes of the affected and the contralateral hippocampus to identify volumetric differences from the grounds of the seizure focus. For this we stablished the affected hippocampus by ictal seizure onset lateralization through vEEG study. Comparisons were performed according to the type of epilepsy (GAD-TLE and niTLE) and compared with HC using Mann Whitney test or Kruskal–Wallis test.

**2.** Hippocampal asymmetry subfields to explore the hypothesis of potential bilateral involvement that could be present in immune epilepsy compared to more circumscribed lesions in other etiology types of epilepsy.

**3.** Cognition: hippocampal subfields volumes were correlated to duration of epilepsy and to neuropsychological memory performance variance. These were performed using the Spearman coefficient. No covariable was used in volumetric analysis for sample size restrictions.

**Results:**

**Volumetry**

In table 1 we resumed a three-way Kruskal-Wallis test comparing each subfield volume on each group of subjects by side.

**Table 1: Hippocampal subfields volumes in cm3 by side (left or right) and group of patients.**

|  | **HC (8)** | | | **GAD65TLE (8)** | | | **niTLE (8)** | | | **P bonferroni** | | |
| --- | --- | --- | --- | --- | --- | --- | --- | --- | --- | --- | --- | --- |
|  | Me | IQR | CV | Me | IQR | CV | Me | IQR | CV | HC  vs GAD65 | HC  vs niTLE | GAD65 vs niTLE |
| **Left** | | | | | | | | | |  |  |  |
|  |  |  |  |  |  |  |  |  |  |  |  |  |
| Parasubiculum | 0.04 | 0.01 | 0.19 | 0.04 | 0.01 | 0.24 | 0.036 | 0.015 | 0.22 | 0.452 | 0.657 | 0.985 |
| Presubiculum | 0.198 | 0.04 | 0.12 | 0.188 | 0.05 | 0.16 | 0.183 | 0.04 | 0.17 | 0.825 | 0.344 | 0.825 |
| Subiculum | 0.27 | 0.07 | 0.13 | 0.27 | 0.08 | 0.18 | 0.22 | 0.08 | 0.25 | 0.643 | 0.120 | 0.643 |
| CA1 | 0.42 | 0.08 | 0.12 | 0.40 | 0.11 | 0.16 | 0.33 | 0.13 | 0.23 | 0.808 | 0.142 | 0.526 |
| CA2-3 | 0.12 | 0.02 | 0.10 | 0.13 | 0.03 | 0.15 | 0.09 | 0.05 | 0.23 | 0.991 | 0.216 | 0.132 |
| CA4 | 0.16 | 0.016 | 0.08 | 0.17 | 0.03 | 0.14 | 0.12 | 0.05 | 0.24 | 0.969 | 0.149 | 0.306 |
| GC-ML-DG | 0.19 | 0.02 | 0.08 | 0.19 | 0.04 | 0.14 | 0.14 | 0.06 | 0.23 | 0.982 | 0.161 | 0.289 |
| Molecular layer | 0.38 | 0.07 | 0.10 | 0.36 | 0.09 | 0.16 | 0.29 | 0.11 | 0.24 | 0.861 | 0.108 | 0.378 |
| HATA | 0.04 | 0.006 | 0.11 | 0.04 | 0.01 | 0.09 | 0.039 | 0.008 | 0.249 | 0.409 | 0.828 | 0.881 |
| Fimbria | 0.06 | 0.01 | 0.18 | 0.05 | 0.02 | 0.25 | 0.05 | 0.014 | 0.25 | 0.653 | 0.384 | 0.965 |
| Hippocampal tail | 0.372 | 0.065 | 0.198 | 0.372 | 0.050 | 0.109 | 0.280 | 0.109 | 0.228 | 0.988 | 0.189 | 0.313 |
| Hippocampal fissure | 0.08 | 0.03 | 0.21 | 0.09 | 0.02 | 0.13 | 0.08 | 0.026 | 0.21 | 0.961 | 0.999 | 0.920 |
| Whole Hippocampus | 2.24 | 0.31 | 0.09 | 2.2 | 0.5 | 0.15 | 1.8 | 0.64 | 0.22 | 0.861 | 0.108 | 0.378 |
| **Right** | | | | | | | | | |  |  |  |
| Parasubiculum | 0.036 | 0.01 | 0.15 | 0.037 | 0.008 | 0.12 | 0.041 | 0.02 | 0.25 | 0.638 | 0.546 | 0.999 |
| Presubiculum | 0.19 | 0.05 | 0.16 | 0.20 | 0.04 | 0.12 | 0.18 | 0.04 | 0.17 | 0.863 | 0.365 | 0.807 |
| Subiculum | 0.28 | 0.05 | 0.23 | 0.28 | 0.025 | 0.1 | 0.28 | 0.07 | 0.21 | 0.996 | 0.943 | 0.987 |
| CA1 | 0.42 | 0.07 | 0.10 | 0.43 | 0.44 | 0.07 | 0.43 | 0.12 | 0.22 | 0.996 | 0.943 | 0.987 |
| CA2-3 | 0.147 | 0.02 | 0.121 | 0.155 | 0.034 | 0.176 | 0.140 | 0.04 | 0.20 | 0.993 | 0.981 | 0.823 |
| CA4 | 0.18 | 0.02 | 0.08 | 0.176 | 0.017 | 0.08 | 0.173 | 0.05 | 0.21 | 0.517 | 0.109 | 0.736 |
| GC-ML-DG | 0.21 | 0.02 | 0.09 | 0.20 | 0.02 | 0.08 | 0.20 | 0.05 | 0.21 | 0.990 | 0.817 | 0.647 |
| Molecular layer | 0.393 | 0.06 | 0.09 | 0.395 | 0.04 | 0.06 | 0.381 | 0.11 | 0.21 | 0.999 | 0.739 | 0.800 |
| HATA | 0.04 | 0.003 | 0.13 | 0.04 | 0.009 | 0.12 | 0.04 | 0.014 | 0.27 | 0.929 | 0.960 | 0.696 |
| Fimbria | 0.05 | 0.01 | 0.23 | 0.05 | 0.009 | 0.14 | 0.06 | 0.02 | 0.36 | 0.919 | 0.891 | 1.000 |
| Hippocampal tail | 0.372 | 0.065 | 0.198 | 0.373 | 0.049 | 0.109 | 0.280 | 0.109 | 0.228 | 0.954 | 0.547 | 0.280 |
| Hippocampal fissure | 0.09 | 0.03 | 0.19 | 0.08 | 0.02 | 0.18 | 0.09 | 0.01 | 0.13 | 0.783 | 0.990 | 0.918 |
| Whole Hippocampus | 2.38 | 0.30 | 0.09 | 2.38 | 0.33 | 0.07 | 2.29 | 0.068 | 0.205 | 0.999 | 0.857 | 0.803 |

Me: median; IQR: interquartile range; CV: coefficient of variation.

In Figure 1S the representation of volume z-score deviations from mean hippocampal volumes of HC is found. There is a non-significant tendency to lower volumes in some subfields of the contralateral hippocampus in GAD-TLE compared to niTLE which shows predominantly positive z-scores in the contralateral hippocampus.

**Figure 1.** **Volume deviation from mean values of HC in both affected and contralateral hippocampus of each TLE group.**

**Asymmetry index:**

GAD-TLE and HC showed no significant differences according to AI:

parasubiculum 7.64 (IQR 8.79) vs 4.24 (IQR 7.24) [t=0.73, p=0.856], presubiculum 7.08 (IQR 8.66) vs 2.56 (IQR 2.76) [t=1.52, p=0.373], subiculum 3.56 (IQR 5.95) vs 3.79 (IQR 3.50) [t=0.46, p<0.0001], CA1 3.47 (IQR 4.45) vs 4.55 (IQR 3.71) [t=0.17, p=0.998],  CA3 6.81 (IQR 13.67) vs 6.82 (IQR 7.24) [t=0.04, p=1.000], CA4  3.29 (IQR 9.90) vs 2.79 (IQR 5.43) [t=0.44, p=0.961], GM-ML-DG 3.52 (IQR 8.96) vs 2.85(IQR 4.92) [t=0.56, p=0.928], HATA 4.35 (IQR 5.04) vs 6.43 (IQR 6.39) [t=0.15, p=0.998], fimbria   6.34 (IQR 8.62) vs 4.37 (IQR 11.51) [t=0.07, p=1.000], molecular layer 4.17 (IQR 11.18) vs 1.59 (IQR 3.80) [t=1.55, p=0.360], hippocampal fissure 4.93 (IQR:7.83) vs 5.21 (IQR 4.70) [t=0.24, p=0.993] and hippocampal tail 4.77 (IQR 5.96) vs 3.04 (IQR 4.13) [t=1.42, p=0.431].

niTLE showed significant asymmetry compared to HC in the following subfields:

Presubiculum  11.96 (IQR 16.9) vs 2.56 (IQR 2.76) [ t=3.42, p=0.008], subiculum 17.13 (IQR 9.16) vs 3.79 (IQR 3.5) [t=4.80, p<0.0001], CA1 21.50 (IQR 11.10) vs 4.55 (IQR 3.71) [t=5.46, p<0.0001],  CA3 19.47 (IQR 13.53) vs 6.82 (IQR 7.24) [t=2.81, p= 0.031], CA4 20.96 (IQR 4.58) vs 2.79 (IQR 5.43) [t=5.39, p<0.0001], GM-ML-DG 20.22 (IQR 4.29) vs 2.85 (IQR 4.92) [t=5.46, p<0.0001],  molecular layer 18.34 (IQR 12.71) vs 1.58 (IQR 3.80) [t=6.10, p< 0.0001] and hippocampal tail 16.76 (IQR 8.77) vs 3.04 (IQR 4.13) [ t= 4.96, p<0.0001].

**Neuropsychology**

Verbal learning encoding as well as delayed recall was similarly impaired in both GAD-TLE and in niTLE despite the side of the epileptic focus. The lateralization of epilepsy followed the general tendency for lower logical memory (ML) scores in left TLE and lower visual memory scores in right TLE. Bilateral GAD-TLE showed the lowest scores. Median z-score for verbal encoding in left GAD-TLE was -1.58 vs -1.85 in niTLE patients. Delayed recall z-score was similarly affected in GAD-TLE and niTLE: -1.37 vs -1.62 but showing slightly higher scores in niTLE. Logical memory evaluation of episodic memory showed minimally lower scores in left niTLE compared with GAD-TLE (41.5 vs 45) and the lowest scores in bilateral GAD-TLE. ML II to assess long-term narrative memory showed the same tendency. Delayed visual evaluation (MVII) was more impaired in right niTLE and showed lower scores in niTLE except for bilateral GAD-TLE (Table 2).

Based on AVLT scores we classified the degree of impairment following these categories: severe impairment if score is less than 45, moderate impairment between 46 and 53, and mild impairment between 54 and 63. We found severe retrieval impairment in 2 GAD-TLE patients and 2 niTLE; moderate retrieval impairment in 5 GAD-TLE patients and 4 niTLE and mild retrieval impairment in 1 GAD-TLE and 2 niTLE patients.

Spearman’s correlation analyses (see table 3) found no relationship between adjusted average hippocampal subfield volume and scores for immediate verbal learning, delayed recall, logical memory and visual memory in all patients.

**Table 2: Median scores performance in different neuropsychological evaluations.**

| Neuropsychological test  median score (p25-p75) | GAD | | niTLE | |  |
| --- | --- | --- | --- | --- | --- |
|  | Left TLE | Right TLE | Left TLE | Right TLE | p |
| Intelligence^1^ | 50(45.5-55) | | 45 (41.5-50) | | NS |
| Logical memory^1^ | 45 (36.5-48.5) | 40 (27-53) | 41.5 (37-45) | 40 (0) | NS |
| Visual memory | 40 (27-53) | 40 (30-50) | 40 (37-43) | 37.5 (20-55) | NS |
| Logical memory II | 45 (35-50) | 43.5 (37-50) | 40 (37-43) | 46 (45-47) | NS |
| Visual memory II | 43 (40-51.5) | 40 (37-43) | 41.5 (40-47) | 37.5 (20-55) | NS |
| Verbal encoding | 41 (45-35) | | 34.5(30-39.5) | | NS |
| Verbal retrieval | 7 (3.5-7.5) | | 5.5 (4.5-7.5) | | NS |
| Executive function | 50 (38-51.5) | | 40 (35-53.5) | | NS |
| Language | 48 (27.5-57) | | 48.5 (44-50) | | NS |
| Attention | 53 (40-53) | | 40 (33-43) | | NS |

**Table 3. Correlation between subfields and verbal learning, verbal and visual memory.** Cognition evaluation: correlation analysis: hippocampal subfields volumes were correlated to neuropsychological memory performance. In GAD-TLE patients we found no significant correlation between any hippocampal subfield volume and logical memory, verbal encoding or retrieval scores.

|  | **Verbal Encoding** | | **Verbal retrieval** | | **ML I** | | **MLII** | |
| --- | --- | --- | --- | --- | --- | --- | --- | --- |
|  | L-GAD65TLE | L-NI TLE | L-GAD65TLE | L-niTLE | L-GAD65TLE | niTLE | L-GAD65TLE | niTLE |
|  | Rho (p) | | | | | | | |
| Parasubiculum | 0.35(0.40) | 0.26(0.53) | 0.09 (0.82) | -0.10(0.82) | 0.24 (0.57) | 0.27 (0.52) | 0.14(0.74) | 0.81 (0.02*) |
| Presubiculum | -0.26 (0.53) | -0.05 (0.91) | -0.43(0.28) | -0.11(0.80) | -0.24 (0.57) | 0.02 (0.95) | -0.24 (0.57) | 0.65 (0.08) |
| Subiculum | -0.16 (0.71) | 0.02 (0.95) | -0.26(0.52) | -0.03(0.93) | -0.31 (0.46) | -0.12 (0.77) | -0.05(0.91) | 0.57 (0.14) |
| CA1 | 0.02 (0.95) | -0.05 (0.91) | -0.08(0.84) | -0.14(0.73) | 0 (1 ) | -0.05 (0.91) | 0.12(0.78) | 0.43 (0.28) |
| CA2-3 | -0.46 (0.25) | -0.1 (0-82) | -0.36(0.38) | -0.02(0.95) | -0.07 (0.87) | -0.05 (0.91) | -0.07(0.87) | 0.26 (0.52) |
| CA4 | -0.24 (0.56) | 0.09 (0.82) | -0.35(0.40) | -0.04(0.93) | -0.29 (0.49) | -0.07 (0.86) | -0.12(0.78) | 0.43 (0.28) |
| GC-ML-DG | -0.13 (0.75) | 0.09 (0.82) | -0.19(0.65) | -0.04(0.93) | -0.21 (0.61) | -0.07 (0.86) | 0.05 (0.91) | 0.43 (0.28) |
| Mol. layer | -0.24 (0.56) | -0.19 (0.65) | -0.35(0.40) | -0.13(0.76) | -0.29 (0.49) | -0.10 (0.82) | -0.12 (0.78) | 0.46 (0.25) |
| HATA | 0.06 (0.89) | -0.17 (0.69) | -0.19(0.65) | 0.11(0.80) | -0.33 (0.42) | -0.37 (0.37) | -0.14(0.73) | 0.01 (0.97) |
| Fimbria | 0.63 (0.09) | -0.1(0.82) | 0.36(0.38) | -0.02(0.95) | 0.19 (0.65) | -0.46 (0.25) | 0.33 (0.42) | -0.44 (0.28) |
| Hippocampal. tail | -0.25 (0.54) | -0.19 (0.65) | -0.32(0.43) | -0.13(0.76) | -0.24 (0.57) | -0.10 (0.81) | -0.09 (0.82) | 0.46 (0.25) |
| Hippocampal fissure | -0.14 (0.73) | 0.09 (0.82) | -0.34(0.41) | -0.13(0.76) | -0.26 (0.53) | -0.07 (0.86) | -0.21 (0.61) | 0.53 (0.18) |
| Whole hippocampus | -0.24 (0.56) | -0.19(0.65) | -0.35(0.39) | -0.13(0.76) | -0.29 (0.49) | -0.10 (0.82) | -0.12 (0.78) | 0.46 (0.25) |

|  | **Verbal Encoding** | | **Verbal retrieval** | | **MV I** | | **MVII** | |
| --- | --- | --- | --- | --- | --- | --- | --- | --- |
|  | R GAD65TLE | R niTLE | R GAD65TLE | R niTLE | R GAD65TLE | R niTLE | R GAD65TLE | R niTLE |
|  | Rho (p) | | | | | | | |
| Parasubiculum | 0.41 (0.31) | 0.50 (0.21) | 0.16 (0.71) | 0.77(0.03*) | 0.25(0.55) | -0.06(0.90) | -0.025(0.95) | 0.06(0.90) |
| Presubiculum | 0.10 (0.82) | 0.14 (0.74) | -0.16 (0.71) | 0.66 (0.08) | 0 (1) | 0.14(0.73) | -0.11 (0.79) | -0.16 (0.71) |
| Subiculum | 0.06 (0.89) | 0.38 (0.35) | -0.19(0.65) | 0.80 (0.02*) | -0.20 (0.63) | 0.26 (0.53) | -0.25 (0.56) | 0.23 (0.59) |
| CA1 | 0.24 (0.56) | 0.23 (0.57) | 0.08(0.84) | 0.80(0.02*) | 0.04 (0.93) | 0.37(0.36) | 0.07 (0.86) | 0.12(0.77) |
| CA2-3 | -0.12 (0.78) | 0.12 (0.79) | 0.01(0.98) | 0.63(0.09) | 0.09 (0.82) | 0.49 (0.22) | 0.38 (0.35) | 0.21 (0.62) |
| CA4 | -0.30 (0.45) | 0.12 (0.79) | -0.29(0.5) | 0.63(0.09) | 0.18 (0.67) | 0.49 (0.22) | 0.07 (0.86) | 0.25 (0.63) |
| GC-ML-DG | -0.26 (0.52) | 0.12 (0.78) | -0.29(0.49) | 0.63(0.09) | -0.08 (0.84) | 0.49 (0.22) | 0.02(0.95) | 0.21(0.63) |
| Mol. layer | 0.06 (0.88) | 0.02 (0.95) | -0.19(0.65) | 0.65(0.08) | -0.20 (0.63) | 0.47(0.24) | -0.24 (0.56) | 0.06 (0.89) |
| HATA | 0.26 (0.52) | 0.29 (0.49) | 0.35(0.40) | 0.50(0.20) | 0.49 (0.22) | 0.53 (0.18) | 0.43 (0.29) | 0.34 (0.41) |
| Fimbria | 0.46 (0.25) | 0.33 (0.42) | 0.26(0.52) | 0.62(0.10) | 0.31(0.45) | 0.28(0.51) | 0.09 (0.84) | 0.12(0.78) |
| Hippocampal tail | -0.22 (0.61) | 0.14(0.74) | -0.16(0.71) | 0.62(0.09) | 0.08 (0.84) | 0.59 (0.13) | 0.18 (0.66) | 0.41 (0.31) |
| Hippocampal fissure | -0.19 (0.65) | 0.79 (0.02*) | -0.08(0.84) | 0.55 (0.16) | 0.18 (0.67) | -0.01(0.98) | 0.18(0.66) | 0.41(0.31) |
| Whole hippocampus | -0.05 (0.91) | 0.09 (0.82) | -0.23(0.59) | 0.71(0.05) | -0.09(0.82) | 0.47(0.24) | -0.20(0.64) | 0.17(0.69) |
